# Alfred, the Zoo Gorilla

**Published:** 1948

**Authors:** R. C. Clarke


### Alfred, the Zoo Gorilla

ALFRED came to the Zoo in September, 1930. He was then thought to be two years old on account of his size, and such evidence as was obtained. As no gorilla had been kept in captivity in England for long, plans were made for his nurture. They consisted of :

1. *Diet.*—This was a difficulty. The normal food for a gorilla is the tops of bamboo shoots and wild celery, which means an enormous amount of roughage : a male gorilla is said to pass 1 cwt. of faeces a day. The Zoo diet arranged for him was milk, special wholemeal bread, and bananas (he was having forty-eight a day at the outbreak of war); and when bananas were no longer obtainable they were replaced by potatoes, lightly boiled in their skins. He also ate such fruit and vegetables as were in season. This was quite successful.

2. *Avoidance of human droplet, and fly-borne infections.* This was done by the construction of a glass screen inside the house and the removal of the barrier to 12 ft. from the outside cage. A fly-proof pantry was built. The keeper fed him by hand, and no food was left about.

3. *Fresh Air and Vitamins.*—Mountain gorillas come from the Kivu Mountains, which are 12,000 ft. in height, on the Equator. It was realized that he was probably used to very cold nights, so he was kept in the fresh air summer and winter, and slept in a very moderately heated house. With such black skin as he had, it was obvious that the sunlight of Bristol could not penetrate it, so he was given fifteen drops of radiostoleum daily.

DEVELOPMENT.—On arrival he weighed 25 lb. He was 45 lb. at the end of the first year and 90 lb. at the end of the second. He was too strong to weigh after that. When he was full-grown, a weighing device was put in his cage; this was difficult to read, but he was thought to be about 350 lb. When he came, the upper central incisors were missing, and these permanent teeth were cut at the age of six. In 1939, at ten years of age, he developed the pad of fibrous tissue on the head—a male secondary sexual characteristic—and it was inferred that puberty started about this time. The large supraorbital ridges developed at the same time as the tuft on the head, but took longer to reach full development. He did not seem to grow or develop after attaining the age of fourteen years.

The external genitals of the male gorilla are remarkably unobtrusive ; a fact which has led to mistaking males for females in the past. Alfred's penis was only seen once when young. It was then about 3 cm. long when erect and the thickness of a slate pencil. No further observation could be made in life and the remark of a keeper who had been with him for fifteen years to the writer, who called to see the corpse soon after death : " He was a male, sir, after all," is illuminating. Examined after death the penis was 8 cm. long with a 3 cm. protrusion and 1.5 cm. in diameter. The scrotum was very shallow ; the gorilla, like many other primates, is able to withdraw his testicles well up under the skin of the abdomen in moments of danger and activity. The testicles weighed 15 gm.

ILLNESSES.—At the age of four, when there was a whooping-cough epidemic in Bristol, he undoubtedly caught it. He would cough hard and often for two or three minutes at a time, but he rapidly recovered. He had never coughed before, nor since, until his final illness. An interesting side-line on this is that droplet infection from whooping-cough carries 12 ft. in the open air. In the summer of 1942 he became very heavy and inactive, and on going into his diet it was found that he was having 12,000 calories a day. This was cut down to 7,000 ; he then became less obese and more healthy and active. In the winter of 1944 he became rather sluggish, fatter, off his food, and his hair came out. Also he suddenly seemed to feel the cold and insisted upon covering himself with sacks. Thyroid deficiency was diagnosed, and thyroid, gr. v., were given him daily. In six weeks he was quite normal, and the thyroid was cut down to gr. ii a day ; this dose was continued for the rest of his life.

At the post-mortem the thyroid gland weighed 18 gm. against an average human weight of 30 gm.—somewhat variable. The whole gland showed considerable fibrosis, with very little functioning gland tissue left.

Last October (1947) he became thin and inactive, with a dry cough. Generalized tuberculosis was diagnosed, but never proved bacteriologically ; he died from this disease in an advanced state. The source of infection was probably carried by keeper's boots from the slaughter-house, where many tuberculous cows have been slaughtered of late. After death he weighed only 224 lb. It was then possible to measure him—his standing height was 5 ft. 11 in. and his arm span was 9ft. 6 in. The skin will be set up by Rowland Ward for the Museum, and the body is preserved in the Anatomy Department for anatomical research.

R. C. CLARKE

